# An Incidental Finding of a Cecal Lymphangioma Managed Conservatively

**DOI:** 10.7759/cureus.60808

**Published:** 2024-05-21

**Authors:** Richard Mitchell, Jonathan Reyes, Vennis Lourdusamy, Raghav Bansal

**Affiliations:** 1 Internal Medicine, Icahn School of Medicine at Mount Sinai/New York City (NYC) Health + Hospitals-Elmhurst, Queens, USA; 2 Gastroenterology, Mount Sinai South Nassau/Icahn School of Medicine at Mount Sinai, Oceanside, USA; 3 Gastroenterology, Bayhealth Medical Sussex Campus, Milford, USA; 4 Gastroenterology, Icahn School of Medicine at Mount Sinai/New York City (NYC) Health + Hospitals-Elmhurst, Queens, USA

**Keywords:** colonoscopy, eus, endoscopy, cecal lymphangioma, colonic lymphangioma

## Abstract

Colonic lymphangiomas are rare and mostly incidental findings found on colonoscopy. It is important to be able to differentiate them from other lesions, such as lipomas. Furthermore, when in close proximity to the appendiceal orifice, such as cecal lesions, they must be differentiated from mucocele and carcinoid tumors. We present a case of a cecal lymphangioma that was managed conservatively. By using endoscopic ultrasound (EUS) and computed tomography to better characterize the cecal mass, it was unnecessary to perform a biopsy or polypectomy. This case highlights that biopsy and/or removal of lymphangiomas are not warranted in all cases of colonic lymphangiomas, especially when lesions are less than 2 cm.

## Introduction

Colonic lymphangioma is a rare finding and is most often an incidental finding during routine colonoscopy. Reporting of colonic lymphangiomas has been increasing, but the reported incidence is still around less than 1 in 20,000 [1,2]. Lesions appear smooth and cystic and commonly contain enlarged lymphatic ducts. The most common sites of origin tend to be the ascending and transverse colons [2]. Cecal lymphangiomas are often solitary and are usually benign in nature. For proper diagnosis and management, it is important to distinguish lymphangiomas from other lesions that affect the cecum. We present a case of a cecal lymphangioma found on a routine screening colonoscopy. In the report, we discuss the modalities used to differentiate a cecal lymphangioma and argue in favor of conservative management.

## Case presentation

**Figure 1:**
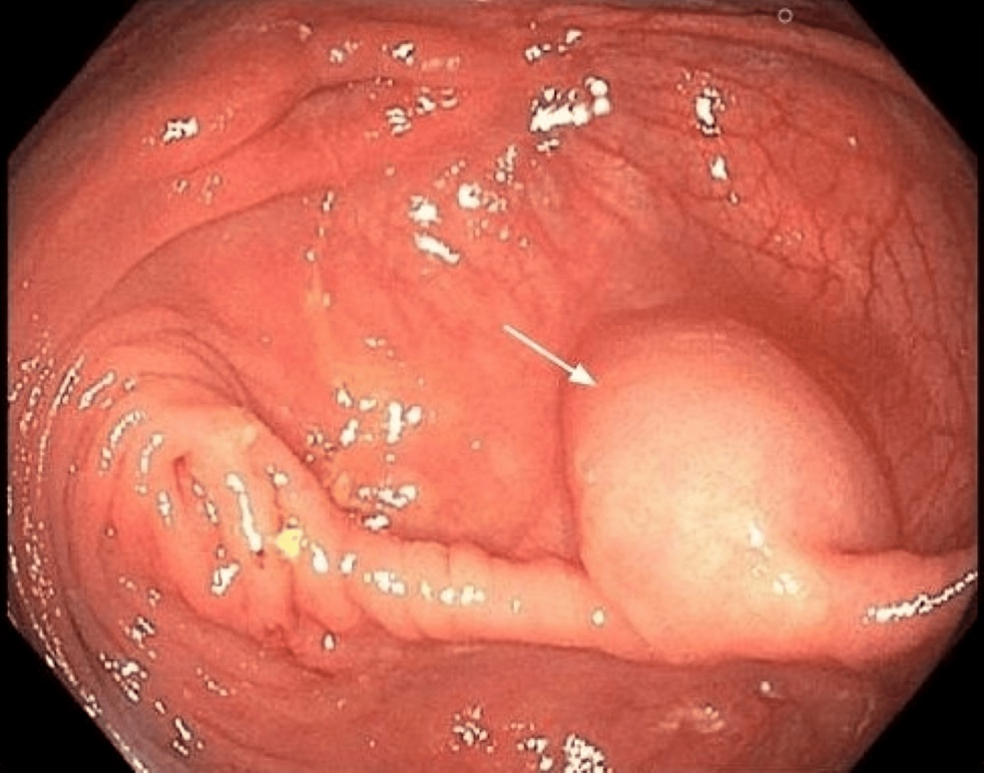
Cecal mass Soft cystic lesion in the cecum measuring approximately 2 cm. White arrow indicates mass.

**Figure 2:**
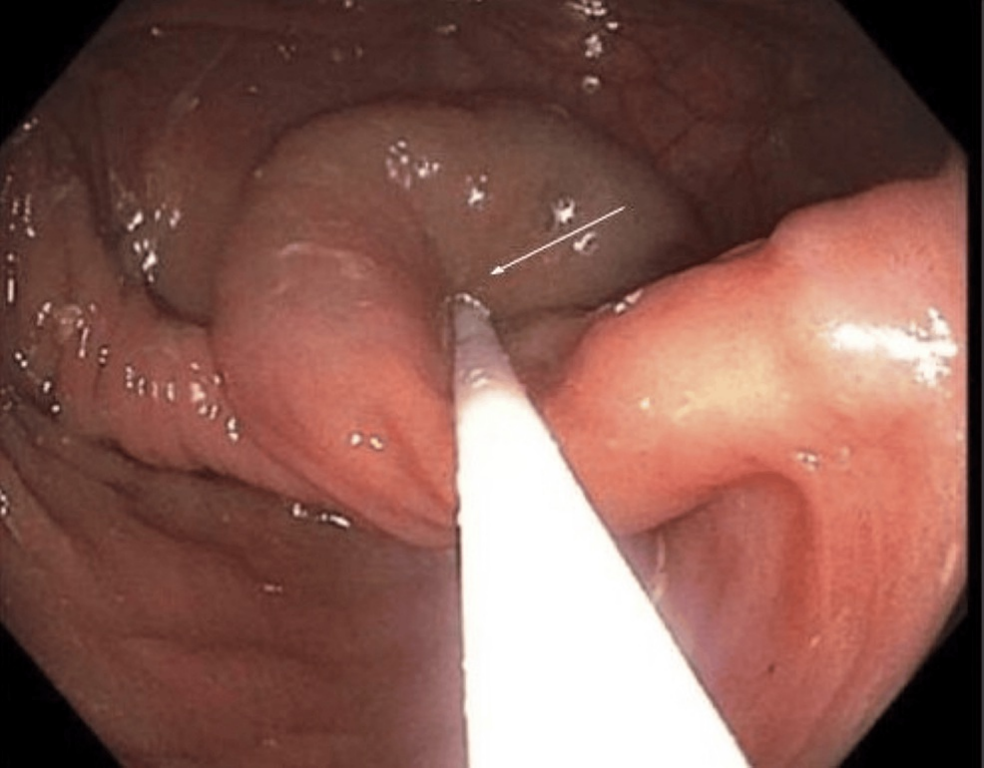
Positive cushion sign Positive cushion sign on endoscopy showing that the mass was freely mobile. White arrow points to the mass being compressible.

**Figure 3:**
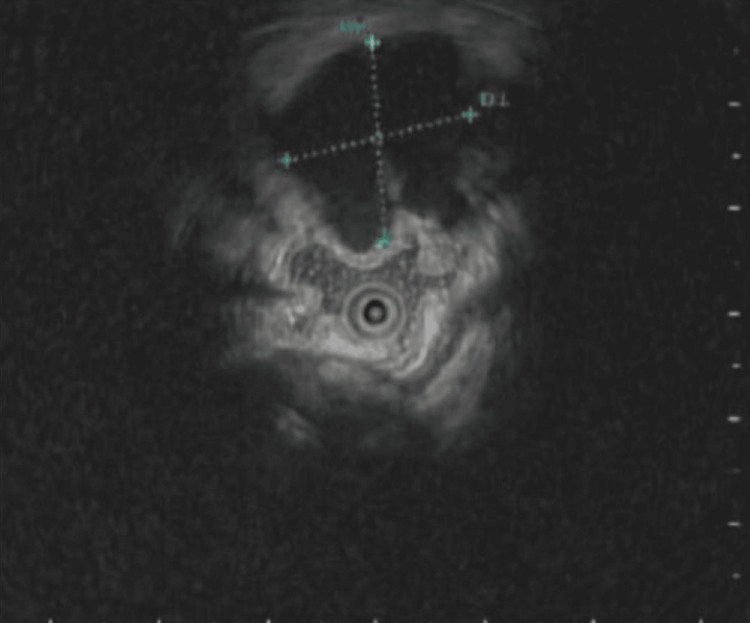
Endoscopic ultrasound Endoscopic ultrasound showing cystic anechoic lesion arising from the submucosa. The lesion measured 1.8 cm × 1.7 cm.

**Figure 4:**
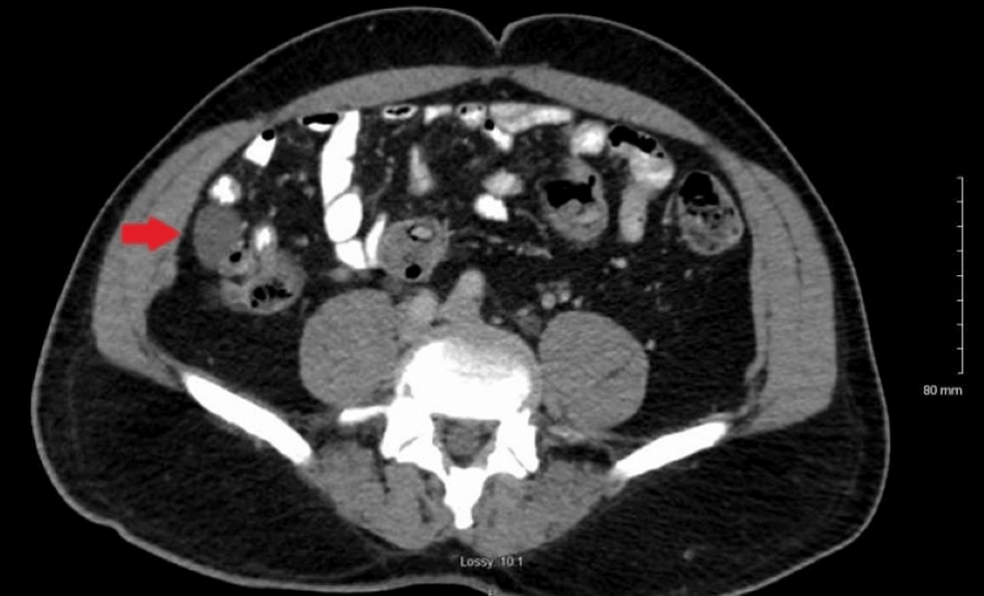
CT of cecal mass Low-density cecal mass on computed tomography measuring 17 Hounsefield units consistent with density of fluid. Red arrow indicated mass.

A 61-year-old male with no significant past medical history was referred by his primary care provider for a routine screening colonoscopy. He denied any symptoms such as abdominal pain, bloody stools, diarrhea, or weight loss. After adequate bowel preparation, a colonoscopy was performed and was significant for a soft cystic mass in the cecum measuring approximately 2 cm (Figure 1). The lesion was freely mobile and was compressible with biopsy forceps (positive for the cushion sign) (Figure 2). The ileocecal valve and appendiceal orifice appeared normal. A mini ultrasound probe exam via the working channel was performed to better characterize the lesion. Ultrasound revealed a 1.8 cm × 1.7 cm cystic anechoic subepithelial lesion with septations arising from the submucosa (Figure 3). The Doppler study did not indicate any evidence of vascular abnormalities. The patient’s prior imaging was reviewed. Computed tomography of the abdomen/pelvis (CTAP) showed a low-density (average 17 Hounsefield units) cystic lesion arising from the wall of the cecum (Figure 4). The patient was asymptomatic with a lesion size of less than 2cm; thus, removal was deemed unnecessary. To the best of our knowledge, our patient has remained asymptomatic with no complications. Thus, follow-up imaging and colonoscopy were not performed.

## Discussion

Colonic lymphangioma was first described by Chisholm and Hillkowitz in 1932 [3]. It is a benign submucosal tumor characterized by its smooth, round, cystic appearance with enlarged lymphatic ducts. After the development and subsequent widespread use of colonoscopy, lymphangioma of the colon became more frequently reported. However, they are still extremely rare in the adult population [1]. Lymphangiomas can occur anywhere in the colon. The most common locations are the ascending and transverse colons, followed by the cecum, with the majority being single lesions [2].

Most patients are asymptomatic, as was the case with our patient. When symptoms are present, they are nonspecific and include lower abdominal pain and constipation [2]. A few case reports have reported intussusception, massive gastrointestinal bleeding, and protein-enteropathy as complications with larger lymphangiomas [4-7].

For diagnostic purposes, lymphangiomas should be differentiated from other lesions. When close to the appendiceal orifice, cecal lymphangiomas should be differentiated from mucoceles and appendiceal cancers such as adenocarcinomas and gastrointestinal neuroendocrine tumors. In our patient, the presence of a normal-appearing appendiceal orifice eliminated the suspicion of a mucocele. CTAP is a useful modality for distinction. Lipomas appear as well-defined fatty masses with a typical fat density that is between −70 and −120 Hounsfield units [8]. In contrast, a lymphangioma appears as a well-defined, multilocular cystic mass with Hounsefield units corresponding to fluid density (−10 to 20) [8,9]. The cecal mass of our patient had an average Hounsefield unit of 17, which was consistent with the density of the fluid. Endoscopic ultrasound (EUS) is an additional tool that has been shown to be helpful in the diagnosis of lymphangiomas [9-11]. Lipomas are hyperechoic lesions, whereas lymphangiomas are hypo- or anechoic, often with separations.

Historically, histological diagnosis has been preferred for definite diagnosis [12]. However, with the advancements in EUS, endosonographic features of anechoic cystic spaces with septations and intact muscularis propria have been considered highly diagnostic for lymphangioma of the colon [9-11]. Additionally, biopsy is not without risks. One case report described an occurrence of iatrogenic infection after a cold-forceps biopsy of an incidental colonic cystic lymphangioma [13]. On EUS, this lesion had the classical endosonographic features of a lymphangioma. Fortunately, the patient improved without any significant complications. Later, the same group concluded that the classic features of EUS are sufficient for a non-invasive diagnosis of cystic lymphangioma of the colon [14]. We agree with these conclusions. Our case acts as similar support and emphasizes the conservative approach in asymptomatic patients with classic findings of cystic lymphangioma on EUS.

If patients are asymptomatic, surgical intervention is not necessary. Unfortunately, there are situations where invasive interventions cannot be avoided, like massive bleeds, intussusception, and invasion of lesions [4-6,12].

## Conclusions

The diagnosis of lymphangioma can be made without the need for a confirmatory biopsy when endoscopic and imaging features are classic for lymphangioma. Classic endoscopic features include a smooth, round cystic appearance with a positive cushion sign. On EUS, anechoic cystic spaces without any solid component and with septations are highly diagnostic. As most lymphangiomas are benign, surgical intervention can be avoided in asymptomatic patients and when lesions are small.
